# Maternal pre-pregnancy obesity and offspring hyperactivity-inattention symptoms at 5 years in preterm and term children: a multi-cohort analysis

**DOI:** 10.1038/s41598-022-22750-8

**Published:** 2022-10-28

**Authors:** Courtney Dow, Elsa Lorthe, Laetitia Marchand-Martin, Cédric Galera, Muriel Tafflet, Pierre-Yves Ancel, Marie-Aline Charles, Barbara Heude

**Affiliations:** 1 Inserm, INRAE, Centre for Research in Epidemiology and StatisticS (CRESS), Université Paris Cité, 75004 Paris, France; 2 Unit of Population Epidemiology, Department of Primary Care Medicine, Geneva University Hospitals, 1205 Geneva, Switzerland; 3 Inserm, Bordeaux Population Health Center, UMR 1219, Univ. Bordeaux, 33000 Bordeaux, France; 4 Centre Hospitalier Perrens, Bordeaux, France; 5 Unit on Children’s Psychosocial Maladjustment, Montreal, QC Canada

**Keywords:** Human behaviour, Psychiatric disorders, Risk factors

## Abstract

The objective of this study was to determine the relationship between maternal pre-pregnancy body mass index (BMI) and child hyperactivity-inattention symptoms (HIS) at 5 years, including preterm and term-born children, and to determine whether this association varied with gestational age. Maternal pre-pregnancy BMI and offspring HIS were assessed in 10,898 participants born ≥ 33 weeks of gestation from the ELFE cohort and 2646 children born between 23 and 34 weeks from the EPIPAGE 2 cohort. Reported pre-pregnancy weight (kg) and measured height (m) were collected from mothers at inclusion and used to classify BMI (kg/m^2^). Child HIS were evaluated using the Strengths and Difficulties Questionnaire around 5 years of age. Logistic regression estimated odds ratios (OR) of a high HIS score (≥ 90th percentile) in the ELFE cohort and generalized estimated equations were used in EPIPAGE 2 to account for non-independence of multiple births. As a negative control, paternal BMI was also considered as an exposure of interest in sensitivity analyses. Maternal pre-pregnancy obesity and overweight were associated with child HIS at 5 years in ELFE (adjusted OR [aOR] for obesity 1.27 [1.06, 1.53]; overweight aOR 1.16 [1.00, 1.36]) and pre-pregnancy obesity was associated with high HIS scores in preterm infants of EPIPAGE 2 (aOR 1.48 [1.06, 2.08]). In ELFE, the magnitude of the association increased with decreasing gestational age (interaction *p* = 0.02). High maternal pre-pregnancy BMI is associated with greater likelihood of high HIS scores in both at-term and preterm children at 5 years of age.

## Introduction

Despite the adverse consequences for both the mother and the child accompanying high maternal body mass index (BMI) before and during pregnancy, rates of overweight and obesity in pregnancy continue to rise globally, with estimated 10-year changes in some countries higher than 90%^1^. This leads to increasing concern as high BMI before pregnancy has been associated with numerous obstetric complications including: gestational diabetes, pre-eclampsia, congenital anomalies, and stillbirth^2^. Maternal pre-pregnancy obesity is also associated with several long-term adverse effects for the offspring such as increased risk for obesity, cardiovascular disease and type 2 diabetes^3^. High maternal pre-pregnancy BMI has also been linked to neurodevelopmental and psychiatric disorders in the child such as attention-deficit hyperactivity disorder (ADHD), cognitive impairment, autism spectrum disorder, anxiety, depression, schizophrenia and eating disorders^4^. ADHD is one of the most common neurodevelopmental disorders in childhood and can have negative long-term effects on the children and their families^5^. Children with ADHD have lower educational and professional attainment and poorer mental health^6,7^. Families suffer from the strain of the condition and ADHD contributes to marital dysfunction^6^. Symptoms also frequently persist into adulthood and more than half of these children have other comorbid conditions^6^, making the underlying etiology of ADHD an important avenue of investigation.

Animal models support a causal link between maternal obesity and offspring hyperactivity^8^. Several large cohort studies of children born at term have similarly observed an association between high maternal pre-pregnancy BMI and ADHD or hyperactivity-inattention symptoms (HIS), which can often accurately estimate an ADHD diagnosis outside of a clinical setting^9^. In a previous study conducted in a cohort of French children born at term from the general population, we also reported an association between maternal pre-pregnancy obesity and high HIS score trajectories in children from 3 to 8 years^10^. This association remained robust even after adjustment for numerous factors such as maternal lifestyle, childcare, child sleeping habits and home stimulation. However, the association between maternal obesity and HIS in premature children has been little explored. To our knowledge, there exists only one study limited to children born extremely preterm (< 28 weeks)^11^ despite the fact that children born preterm are already at increased risk of HIS/ADHD^12^ and the risk of neurodevelopmental deficits appear to increase in a step-wise manner with decreasing gestational age^13^. Compared to term-born children, premature children are also at increased risk for infant mortality, neonatal morbidity and numerous neurodevelopmental deficits^13^ that persist into adulthood as long-term disabilities and affect their educational attainment and incomes^14^. As maternal obesity is a state of chronic low-grade inflammation and inflammation has been shown to have adverse effects on the developing brain^4,15^, it is possible that infants born premature, both more functionally and immunologically immature than infants born at term, may be more sensitive to the insults triggered by maternal obesity due to the heightened or dysregulated inflammatory state they may be exposed to both in utero and after birth^16^. Given that maternal obesity is also a major risk factor for preterm labour^17^ and that this population is already highly vulnerable, it is imperative to identify and understand the consequences of modifiable risk factors, such as maternal pre-pregnancy obesity, that can influence a healthy birth and childhood.

Thus, the primary objective of this study was to determine if high maternal pre-pregnancy BMI is associated with child hyperactivity-inattention symptoms in both preterm and at-term infants. Secondarily, we aimed to determine whether this association varied in conjunction with gestational age.

## Methods

### Study population

#### ELFE

The ELFE cohort (French Longitudinal Study since Childhood) was launched in 2011 to study the determinants, from birth and throughout the life course, of child health, development and socialization^18^. Children were recruited at birth from 320 randomly selected maternity units throughout metropolitan France who agreed to participate. This recruitment spanned 25 days in four periods throughout the year. Eligibility criteria included: births ≥ 33 weeks of gestation, mothers ≥ 18 years old, and no plan to leave metropolitan France in the following 3 years. Information and consent documents were available in French, English, Arabic and Turkish and written informed consent was obtained from parents or the mother alone at enrollment. If not present at enrollment, the father was informed of his right to deny participation.

**Figure 1:**
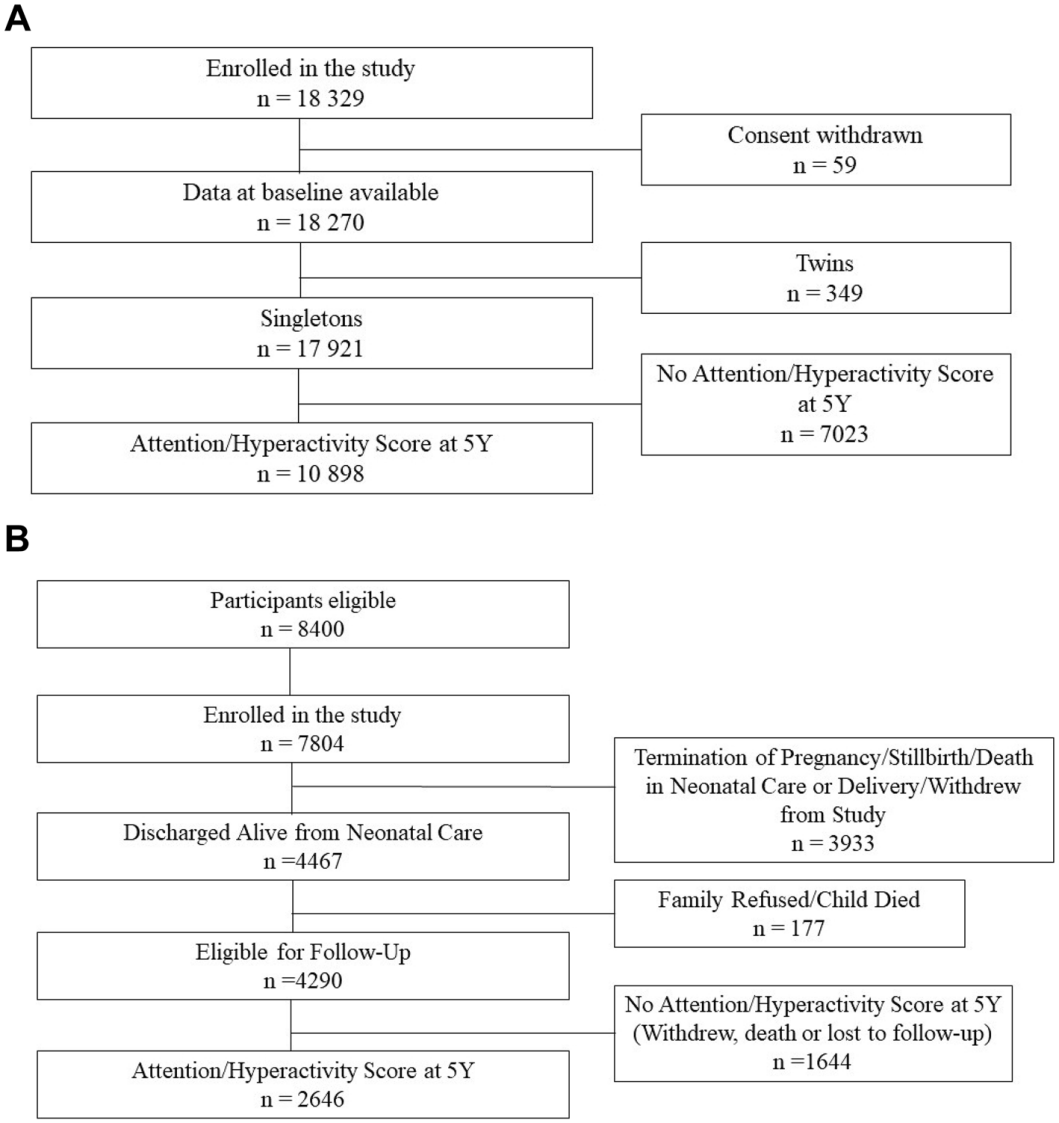
(**A**) Flowchart of study participation in the ELFE cohort study. (**B**) Flowchart of study participation in the EPIPAGE 2 cohort study.

In total, 18,040 mothers agreed to participate in ELFE and gave birth to 18,329 infants (Fig. 1A). For 59 women, baseline data were withdrawn at her demand, resulting in an initial study population of 18,270 children. Data were collected from telephone interviews and internet or paper questionnaires with one or both parents.

#### Epipage 2

EPIPAGE 2 is a large population-based French cohort of preterm infants designed to study the short and long-term outcomes in preterm infants, the determinants of preterm birth, and to examine the impact of the changes in medical care and practices on preterm infants^19^. Live born, still born babies, and terminations of pregnancy (TOP) between 22 and 34 weeks of gestation in all maternities in 25 of 26 French regions were eligible. The recruitment period varied by gestational age group (extremely preterm [22–26 weeks of gestation], very preterm [27–31 weeks], moderately preterm [32–34 weeks]), with longer recruitment periods for the smaller gestational age groups. Between March 2011 and December 2011, 7804 participants were recruited into EPIPAGE 2 (Fig. 1B). A total of 4467 infants were discharged alive from neonatal care and 177 subsequently withdrew (family refused or child died), resulting in a total of 4290 infants eligible for follow-up.

At birth and in the neonatal period, data were extracted from medical records. Follow-up information was collected by questionnaires completed by parents, physicians, and assessments of health status and development at regional exam centres.

### Exposure assessment

Information about maternal pre-pregnancy height and weight were extracted from medical records (EPIPAGE 2) or collected through face-to-face interviews by survey technicians at the time of delivery (ELFE). Maternal pre-pregnancy BMI (kg/m^2^) was subsequently categorized using the World Health Organization classification: underweight (< 18.5), normal weight (18.5–24.9), overweight (25.0–29.9) and obese (≥ 30).

### Outcome assessment

HIS were assessed using the Strengths and Difficulties Questionnaire (SDQ) at 5.5 years of age. In ELFE, the questionnaire was administered through a phone interview with one of the parents (usually the mother), while in EPIPAGE 2 parents responded through a self-administered questionnaire. The SDQ is a validated tool to detect problem behaviours and emotions in children^20^. It includes 5 domains (hyperactivity-inattention, emotional symptoms, conduct problems, peer problems and prosocial behaviour), each comprised of 5 items with responses ranging from “Not true (0 points)” to “A little true (1 point)” to “Very true (2 points)”. The resulting score from each domain can range from 0 to 10. Data on HIS were collected for 11,247 children in ELFE and 2646 in EPIPAGE 2. A score ≥ 90th percentile in ELFE was considered “High”, which corresponded to a score of 7. This cut-off is in agreement with both the traditional SDQ threshold (which predicts a substantially increased probability of an independently diagnosed psychiatric disorder) and the French-specific threshold for “Abnormal”^20,21^. The same threshold was used in EPIPAGE 2.

### Covariates

At baseline, covariates collected included maternal and paternal: age, country of birth, level of education, employment status, region of residence, household income and cohabitation status. Factors collected or calculated related to the pregnancy were: maternity unit, parity, folic acid intake before conception/from 0 to 12 weeks/ > 12 weeks (ELFE only), folic acid supplements around conception (EPIPAGE 2), gestational weight gain (ELFE), type 1 or 2 diabetes history, gestational diabetes, hypertension during pregnancy, pre-eclampsia, any psychiatric disorder before or during pregnancy, mode of delivery, cause of prematurity (EPIPAGE 2), gestational age, birth weight, birth length and maternal anxiety score (EPIPAGE 2). Variables concerning maternal lifestyle: smoking during pregnancy, alcohol consumption during pregnancy (ELFE), diet quality during pregnancy (score of adherence to the National Nutrition guidelines for pregnant women; ELFE)^22^ and physical activity during pregnancy (score = metabolic equivalents (METS)_activity_ × time (hours) × 7 (days); ELFE). Information concerning current paternal anthropometrics (height, weight) was collected in the questionnaire 2 months postpartum (ELFE) or at discharge from the hospital (EPIPAGE 2). In ELFE, the question was directed to the father but could be responded to by the mother if the father did not respond. In EPIPAGE 2, the question was directed to the mother. Lastly, variables related to the child included: sex, duration of breastfeeding, childcare from birth to 5 years, sleep duration at 2 years, difficulties falling asleep at 2 years, duration of daily screentime at 2 years (ELFE), and the HOME-SF (Home Observation Measurement of the Environment-Short Form) score, a measurement of the quality and quantity of stimulation in the child’s home^23^ at 5.5 years (EPIPAGE 2).

### Statistical analysis

#### Missing data

In the complete ELFE cohort (n = 18,329), missing data ranged from 0.3 to 46.3% from birth to 5.5 years (Supplementary Table S1a), while missing data ranged from 0 to 65% in EPIPAGE 2 (n = 4467) (Supplementary Table S1b). Multiple imputation with chained equations was used to impute missing data and generate 45 imputed datasets in ELFE and 65 in EPIPAGE 2. All variables listed above were used in the multiple imputation, with the discriminant function method for categorical variables and predictive mean matching for quantitative variables. To validate the imputed datasets, the means and frequencies for each imputation were compared to the original dataset to determine if they differed significantly^24^. The imputed datasets did not significantly differ from the original dataset (*results not shown*).

#### Weighting

After excluding those for which baseline data could not be imputed in ELFE (n = 59), inverse probability weighting (IPW) was used to attenuate the bias due to attrition in both ELFE and EPIPAGE 2. Models were fit for baseline data associated with dropout before the 5-year follow-up (*p* < 0.20). In ELFE, logistic regression models were used while in EPIPAGE 2 generalized estimating equations (GEE) models allowed us to take into account the large number of multiple births. Stabilized weights were calculated from these models and subsequently applied to further analyses. In general, attrition was more likely in younger parents who were not born in France, with lower socioeconomic status, and mothers with more health problems or an unhealthier lifestyle (Supplementary Table S2).

#### Data analysis

In ELFE, we excluded the small number of twins (n = 349) and those missing HIS data at 5.5 years (n = 7023), the final study population included 10,898 children (Fig. 1A). The final study population in EPIPAGE 2 was 2646 children after excluding those with no HIS data at 5.5 years (Fig. 1B). Descriptive analyses were performed on both populations to describe the overall distribution of characteristics. In EPIPAGE 2, the descriptive statistics were weighted to account for the recruitment scheme. Characteristics of participants with low and high (≥ 90th percentile) HIS scores were compared using ANOVA or by chi^2^ test, as appropriate. As high pre-pregnancy BMIs have been associated with greater risk of perinatal death^25^, we also performed descriptive analyses in the EPIPAGE 2 population between covariates and vital status (termination of pregnancy; stillbirth; death in delivery room; death in neonatology; alive at discharge) to better understand the competing risks in this vulnerable population.

An extensive literature review allowed the identification of potential confounding factors. We performed logistic regression in ELFE and GEE models in EPIPAGE 2 to account for the non-independence of multiple births. Univariate models were performed on all potential confounders and those associated with a high HIS score were considered for inclusion in the multivariate model (*p* < 0.20). Variables were retained in the multivariate model if they were considered essential based on the literature or if they changed the beta coefficients for the BMI categories by more than 10%. We tested for interactions with sex, gestational weight gain (ELFE only), gestational diabetes, hypertension and gestational age. All statistical analyses were performed on SAS 9.4 (SAS Institute Inc, Cary, NC).

### Sensitivity analyses

Sensitivity analyses were conducted: (1) using only complete cases to evaluate the robustness of our findings with respect to the selection bias due to missing data; (2) Using the continuous HIS score; (3) Adjusting for paternal BMI, using it as a negative control of unmeasured familial or genetic confounding; (4) Additionally adjusting for gestational weight gain (ELFE only), as the role of gestational weight gain in the relationship between maternal obesity and child neurodevelopment is not clear; (5) Testing for interactions with sex, gestational weight gain and gestational age (continuous); (6) using the same adjustment factors for models in both ELFE and EPIPAGE 2 (and excluding potential moderators or mediators of the relationship).

### Ethics approval

Both ELFE and EPIPAGE 2 received ethics approval for data collection from the French National Commission on Informatics and Liberty (CNIL), the Advisory Committee on Information Processing in Material Research in the Field of Health (CCTIRS), and the Committee for Protection of People (CPP).

## Results

### Descriptive

The descriptive characteristics of the ELFE study population are summarized in Table 1. Children with higher HIS scores were more likely to have slightly younger mothers with lower education, income, and no previous children. Parents of children with higher HIS scores were more likely to be obese or underweight, and mothers were more likely during pregnancy to smoke, have a psychiatric disorder, and have a less healthy diet. Children with higher HIS scores were more likely cared for by parents rather than in childcare centres, had more difficulties falling asleep at 2 years, longer screentime durations at 2 years and were slightly younger at evaluation.

**Table 1:** Descriptive characteristics of the overall population (N [%] or mean [STD]) by SDQ hyperactivity-inattention score in the ELFE study (n = 10,898).

Variable	SDQ hyperactivity-inattention score			
	Study Population n = 10,898	Score < 7 n = 9645	Score ≥ 7 n = 1253	*p*-value^a^
**Sociodemographics**				
Maternal age (at birth) (n = 10,855)	30.9 [4.7]	31.0 [4.6]	30.1 [4.9]	< 0.001
Maternal Country of birth (n = 10,895)				0.40
France	9861 (90.5)	8715 (90.4)	1146 (91.5)	
EU	234 (2.1)	212 (2.2)	22 (1.8)	
Other	803 (7.4)	718 (7.4)	85 (6.8)	
Maternal education (at birth) (n = 10,893)				< 0.001
High	7378 (67.7)	6666 (69.1)	712 (56.8)	
Medium	3038 (27.9)	2565 (26.6)	473 (37.7)	
Low	480 (4.4)	412 (4.3)	68 (5.4)	
Monthly income (n = 10,402)				< 0.001
1st quartile	2571 (24.7)	2173 (23.6)	398 (33.4)	
2nd quartile	3578 (34.4)	3161 (34.3)	417 (35.0)	
3rd quartile	2534 (24.4)	2285 (24.8)	249 (20.9)	
4th quartile (highest)	1721 (16.5)	1594 (17.3)	127 (10.7)	
**Parental health**				
Maternal BMI category (n = 10,749)				< 0.001
Underweight (< 18.5 kg/m^2^)	755 (7.0)	667 (7.0)	88 (7.1)	
Normal (18.5-25 kg/m^2^)	7252 (67.4)	6492 (68.3)	760 (61.0)	
Overweight (25-30 kg/m^2^)	1774 (16.5)	1530 (16.1)	244 (19.6)	
Obese (≥ 30 kg/m^2^)	971 (9.0)	818 (8.6)	153 (12.3)	
Paternal BMI category (n = 9383)				< 0.001
Underweight (< 18.5 kg/m^2^)	72 (0.8)	63 (0.8)	9 (0.9)	
Normal (18.5-25 kg/m^2^)	5221 (55.6)	4671 (56.0)	550 (52.8)	
Overweight (25-30 kg/m^2^)	3378 (36.0)	3005 (36.0)	373 (35.8)	
Obese (≥ 30 kg/m^2^)	716 (7.6)	607 (7.3)	109 (10.5)	
Parity (n = 10,755)				< 0.001
0	4964 (46.1)	4293 (45.1)	671 (54.0)	
1	3921 (36.4)	3519 (37.0)	402 (32.4)	
2	1405 (13.1)	1280 (13.5)	125 (10.1)	
≥ 3	468 (4.4)	424 (4.5)	44 (3.5)	
Gestational weight gain (kg) (n = 10,682)	13.2 [5.2]	13.2 [5.1]	13.4 [5.7]	0.25
History of diabetes (n = 10,600)				0.09
No	10,241 (96.6)	9078 (96.7)	1163 (95.6)	
Type I/Type II	101 (1.0)	85 (0.9)	16 (1.3)	
Previous gestational diabetes	258 (2.4)	220 (2.3)	38 (3.1)	
Gestational diabetes (n = 10,384)				0.99
No	9674 (93.3)	8567 (93.3)	1107 (93.3)	
Yes	690 (6.7)	611 (6.7)	79 (6.7)	
Hypertension during pregnancy (n = 10,596)				0.08
No	10,386 (98.0)	9205 (98.1)	1181 (97.4)	
Yes	210 (2.0)	178 (1.9)	32 (2.6)	
Any psychiatric disorder (during pregnancy) (n = 10,797)				< 0.001
No	9487 (87.8)	8429 (88.2)	1058 (84.8)	
Yes	1313 (12.2)	1123 (11.8)	190 (15.2)	
**Maternal lifestyle**				
Maternal smoking in pregnancy (n = 10,792)				< 0.001
No	9015 (83.5)	8039 (84.2)	976 (78.2)	
Yes	1780 (16.5)	1508 (15.8)	272 (21.8)	
Maternal alcohol intake in pregnancy (n = 10,153)				0.06
Light (< 3 units)	6103 (60.1)	5370 (59.8)	733 (62.8)	
Moderate (3–7 units)	3967 (39.1)	3545 (39.5)	422 (36.1)	
Heavy (≥ 7 units)	84 (0.8)	71 (0.8)	13 (1.1)	
Pregnancy diet quality score (n = 9796)	7.7 [0.8]	7.7 [0.8]	7.6 [0.8]	< 0.001
Physical activity score (3rd Trimester) (n = 9853)	174.5 [84.7]	173.9 [84.6]	179.0 [85.4]	0.06
**Pregnancy/delivery**				
Gestational age (weeks) (n = 10,586)	39.6 [1.4]	39.6 [1.4]	39.7 [1.4]	0.51
Birth weight (g) (n = 10,532)	3340 [479]	3338 [479]	3352 [479]	0.35
**Child characteristics**				
Duration of any breastfeeding (n = 9094)				0.41
None	2920 (32.1)	2603 (32.3)	317 (31.0)	
< 6 months	4313 (47.4)	3831 (47.5)	482 (47.1)	
≥ 6 months	1861 (20.5)	1636 (20.3)	225 (22.0)	
Sex of child (n = 10,645)				0.60
Male	5522 (51.9)	4879 (51.8)	643 (52.6)	
Female	5123 (48.1)	4543 (48.2)	580 (47.4)	
Childcare at 2Y (n = 10,404)				0.01
Parents	2630 (25.3)	2285 (24.8)	345 (29.0)	
Relatives	779 (7.5)	688 (7.5)	91 (7.7)	
Professional	4595 (44.1)	4091 (44.4)	504 (42.4)	
Childcare Centre	2404 (23.1)	2155 (23.4)	249 (20.9)	
Difficulties falling asleep (2Y) (n = 10,370)				< 0.001
Never	5517 (53.2)	4947 (53.8)	570 (48.3)	
Sometimes	3262 (31.4)	2867 (31.2)	395 (33.4)	
Often	1596 (15.4)	1380 (15.0)	216 (18.3)	
Total screentime duration (hours/day) (2Y) (n = 10,472)	0.41 [0.5]	0.41 [0.5]	0.47 [0.6]	< 0.001
Child's age at 5Y follow-up (months) (n = 10,895)	66.5 [1.8]	66.5 [1.8]	66.3 [1.7]	< 0.001

^a^Difference by hyperactivity-inattention score, by ANOVA or by chi^2^ test.

*BMI* body mass index.

Table 2 displays the descriptive characteristics of the EPIPAGE 2 study population by HIS scores. Children with higher HIS scores were more likely to have younger parents and in very and moderately preterm children, these mothers were more likely from a lower SES class and the children were more likely male. Very preterm children with high HIS were more likely born to mothers from France, with higher BMI, more symptoms of anxiety, and who were less likely to smoke during pregnancy. Very preterm children with HIS scores ≥ 7 were also born with lower birth weight, breastfed for shorter periods of time, had a less stimulating home environment at 5.5 years and suffered more frequent night waking at 2 years.

**Table 2:** Weighted descriptive characteristics of the overall population (N [%] or mean [STD]) and SDQ hyperactivity-inattention score in the EPIPAGE 2 study (n = 2646).

Variable	Study population n = 2646	Hyperactivity-inattention score < 7 n = 2084	Hyperactivity-inattention score ≥ 7 n = 562	*p*-value^a^
**Sociodemographics**				
Maternal age (at birth) (n = 2646)	30.6 [0.1]	30.9 [0.2]	29.3 [0.3]	< 0.001
Maternal Country of birth (n = 2640)				0.24
France	2169 (84.7)	1687 (84.1)	481 (87.2)	
EU	314 (9.9)	267 (10.6)	47 (7.4)	
Other	157 (5.3)	123 (5.3)	33 (5.4)	
Maternal education (birth) (n = 2569)				0.02
High	1351 (54.0)	1107 (55.9)	244 (46.6)	
Medium	528 (20.4)	410 (19.9)	118 (22.6)	
Low	690 (25.6)	509 (24.3)	180 (30.8)	
Household SES (n = 2541)				< 0.001
Mangerial	661 (27.3)	562 (29.7)	99 (17.5)	
Intermediate	632 (25.8)	504 (26.2)	128 (24.1)	
Administrator, company director, civil servant, student	647 (25.2)	501 (24.0)	146 (30.0)	
Domestic or sales employee	309 (11.3)	211 (9.9)	96 (16.8)	
Labourer	249 (8.7)	190 (8.5)	59 (9.7)	
Without profession	43 (1.7)	32 (1.7)	11 (1.9)	
Parity (n = 2622)				0.03
0	1502 (56.8)	1162 (55.2)	340 (63.3)	
1	632 (25.4)	505 (26.1)	127 (22.7)	
≥ 2	488 (17.8)	398 (18.7)	88 (14.0)	
**Parental health/lifestyle**				
Maternal BMI category (n = 2465)				0.10
Underweight (< 18.5 kg/m^2^)	180 (6.7)	136 (6.2)	44 (8.8)	
Normal (18.5-25 kg/m^2^)	1430 (58.8)	1149 (60.2)	281 (53.2)	
Overweight (25-30 kg/m^2^)	488 (20.1)	390 (19.9)		98 (20.9)
Obese (≥ 30 kg/m^2^)	367 (14.3)	264 (13.6)	103 (17.1)	
Paternal BMI category (n = 975)				N/A*
Underweight (< 18.5 kg/m^2^)	5 (0.4)	5 (0.6)	0	
Normal (18.5-25 kg/m^2^)	545 (56.7)	434 (58.1)	111 (51.2)	
Overweight (25-30 kg/m^2^)	323 (30.7)	250 (29.4)	73 (29.4)	
Obese (≥ 30 kg/m^2^)	102 (12.1)	79 (11.9)	23 (13.0)	
Any diabetes in pregnancy (n = 2452)				0.69
No	2201 (88.2)	1728 (88.0)	471 (88.9)	
Yes	251 (11.8)	196 (12.0)	55 (11.1)	
Gestational diabetes (n = 2469)				0.82
No	2256 (90.2)	1771 (90.1)	483 (90.6)	
Yes	213 (9.8)	166 (9.9)	47 (9.4)	
Hypertension during pregnancy (n = 2491)				0.57
No	2079 (85.3)	1641 (85.6)	437 (84.3)	
Yes	412 (14.7)	318 (14.4)	93 (15.7)	
Maternal smoking in pregnancy (n = 2556)				< 0.01
No	2080 (83.2)	1682 (84.6)	397 (77.3)	
Yes	476 (16.8)	334 (15.4)	142 (22.7)	
Anxiety score in pregnancy (STAI-T) (n = 1781)				0.05
Weak	1409 (81.7)	1128 (83.1)	281 (76.3)	
Moderate	222 (11.0)	167 (10.4)	55 (13.3)	
High	150 (7.3)	108 (6.5)	42 (10.4)	
**Pregnancy/delivery**				
Gestational age (n = 2646)				< 0.001
< 27 weeks	329 (4.6)	237 (4.1)	92 (6.5)	0.02
27–31 weeks	1659 (31.2)	1312 (30.7)	347 (32.9)	
≥ 32 weeks	658 (64.3)	535 (65.2)	123 (60.6)	
Birth weight (g) (n = 2646)	1713 [12]	1725 [14]	1663 [30]	0.01
Cause of prematurity (n = 2415)				0.84
Preterm labour	1086 (48.4)	865 (48.6)	221 (47.5)	
Preterm PROM	592 (23.5)	467 (23.8)	125 (22.2)	
Vascular pathology or isolated placental abruption	581 (21.9)	451 (21.5)	130 (23.3)	
Isolated IUGR	156 (6.3)	114 (6.1)	42 (6.9)	
**Child characteristics**				
Duration of breastfeeding (n = 2383)				0.02
None	837 (34.1)	637 (32.8)	200 (39.6)	
< 6 months	1465 (62.7)	1170 (63.6)	295 (59.2)	
≥ 6 months	81 (3.1)	72 (3.5)	8 (1.2)	
Sex of child (n = 2646)				< 0.001
Male	1420 (55.5)	1050 (52.2)	370 (68.8)	
Female	1226 (44.5)	1034 (47.8)	192 (31.2)	
Childcare at 2Y (n = 2490)				0.52
Parents	1022 (38.9)	795 (38.5)	226 (40.6)	
Relatives/employee at home	173 (6.9)	128 (6.5)	45 (8.8)	
Professional/Childcare Centre	423 (18.0)	332 (18.1)	91 (17.5)	
Maternal assistant	626 (27.9)	511 (28.3)	115 (26.4)	
Other	246 (8.2)	198 (8.6)	47 (6.7)	
Night waking (2Y) (n = 2457)				0.02
Never	829 (35.4)	687 (36.7)	141 (30.0)	
Once/night or less	945 (39.3)	761 (39.7)	183 (37.6)	
Twice/night	563 (20.7)	408 (19.4)	155 (26.1)	
≥ Three times/night	120 (4.6)	82 (4.2)	38 (6.3)	
HOME score (n = 2460)	23 [0.1]	23.1 [0.1]	22.6 [0.2]	< 0.01
Child's age at 5Y follow-up (months) (n = 2646)	67.3 [0.1]	67.3 [0.07]	67.5 [0.14]	0.21

Characteristics are weighted using sampling weights that take into account the different periods of recruitment by gestational age group.

^a^Difference by hyperactivity-inattention score, by ANOVA or by chi^2^ test.

*BMI* body mass index, *IUGR* intrauterine growth restriction, *HOME* Home Observation Measurement of the Environment-Short Form, *PROM* premature rupture of the membranes.

*Chi^2^ test statistic not calculated due to the 0 cell.

### Vital status by maternal BMI in EPIPAGE 2

Mothers with pre-pregnancy obesity were over-represented in the vital status categories: stillborn, died in delivery room and died in neonatology (Supplementary Table S3). With regards to stillborns and infants that died in neonatology, women who were underweight before pregnancy were also more prevalent.

### Maternal BMI and hyperactivity-inattention symptoms in offspring

In bivariate logistic regression in ELFE, maternal pre-pregnancy obesity, overweight and underweight were significantly associated with increased odds of a high HIS score in children at 5 years (unadjusted odds ratio [OR] 1.42 [95% CI 1.19, 1.69]; OR 1.28 [1.10, 1.49]; OR 1.27 [1.03, 1.56]; for obesity, overweight, and underweight, respectively) (Table 3). Adjustment for confounding factors attenuated the relationship between maternal obesity and high offspring HIS scores slightly (adjusted OR [aOR] 1.27 [1.06, 1.53]) and those for maternal overweight to the threshold of statistical significance (aOR 1.16 [1.00, 1.36]). After adjustment maternal underweight was no longer statistically significant, (aOR 1.14 [0.92, 1.41]).

**Table 3:** Association between maternal pre-pregnancy body mass index and offspring hyperactivity-inattention symptoms (SDQ score ≥ 7) in ELFE (n = 10,898) and EPIPAGE 2 (n = 2646) at 5.5 years.

Variable	Unadjusted OR [95%CI]	*p*-value	Adjusted OR^a^ [95%CI]	*p*-value
**ELFE**				
Obese (≥ 30 kg/m^2^)	1.42 [1.19, 1.69]	< 0.001	1.27 [1.06, 1.53]	0.01
Overweight (25-30 kg/m^2^)	1.28 [1.10, 1.49]	< 0.01	1.16 [1.00, 1.36]	0.05
Normal (18.5-25 kg/m^2^)	REF		REF	
Underweight (< 18.5 kg/m^2^)	1.27 [1.03, 1.56]	0.03	1.14 [0.92, 1.41]	0.24
**EPIPAGE 2**				
Obese (≥ 30 kg/m^2^)	1.39 [1.02, 1.89]	0.03	1.48 [1.06, 2.08]	0.02
Overweight (25-30 kg/m^2^)	0.91 [0.68, 1.20]	0.50	0.96 [0.72, 1.30]	0.81
Normal (18.5-25 kg/m^2^)	REF		REF	
Underweight (< 18.5 kg/m^2^)	1.29 [0.83, 2.02]	0.26	1.07 [0.69, 1.67]	0.75

^a^Adjusted for maternal age at birth, maternal education, household income (ELFE), household socioeconomic category (EPIPAGE 2), parity, sex, psychological problems during pregnancy (ELFE), anxiety symptoms (EPIPAGE 2), singleton pregnancy (EPIPAGE 2), maternal physical activity during pregnancy (ELFE), maternal healthy diet score during pregnancy (ELFE), maternal alcohol intake during pregnancy (ELFE), maternal smoking during pregnancy, child age at evaluation, gestational age, breastfeeding, cause of prematurity (EPIPAGE 2), childcare at 2 years, screentime at 2 years (ELFE), HOME score (EPIPAGE 2), difficulties falling asleep at 2 years (ELFE) and frequent night waking at 2 years (EPIPAGE 2).

Missing covariates are imputed and weighted using IPW.

In univariate analyses in EPIPAGE 2, maternal obesity was associated with increased odds of a high HIS score (OR 1.39 [1.02, 1.89]), while overweight and underweight were not. After adjustment for covariates, the magnitude of the relationship became slightly stronger (aOR 1.48 [1.06, 2.08]) (Table 3).

### Sensitivity analyses

#### Complete case analyses

In complete case analyses in ELFE, we observed similar, though somewhat stronger in magnitude, relationships between maternal pre-pregnancy obesity and overweight with high HIS scores. Univariate models including 10,640 participants without missing data found comparable estimates to the imputed and weighted datasets (Supplementary Table S4a). After adjustment for confounding factors, the odds ratios were attenuated but remained significantly associated with a high HIS score in a population of 7185 children. Univariate complete case analyses in EPIPAGE 2 included 2465 participants (Supplementary Table S4b). Maternal pre-pregnancy obesity was associated with 1.5× increased odds of a high HIS score in offspring. After adjustment for covariates and the population decreased to 1232, the estimates were attenuated to 1.4x.

#### Linear regression analyses

In adjusted linear models for ELFE, both pre-pregnancy obesity and overweight were strongly associated with HIS scores as a continuous variable (*p* < 0.001 for obese mothers; *p* < 0.001 for overweight mothers) (Supplementary Table S5). Pre-pregnancy underweight was not associated with HIS scores in offspring at 5 years (*p* = 0.35). In adjusted linear models in EPIPAGE 2, only maternal obesity was associated with higher HIS scores (*p* < 0.001).

#### Paternal BMI

**Figure 2:**
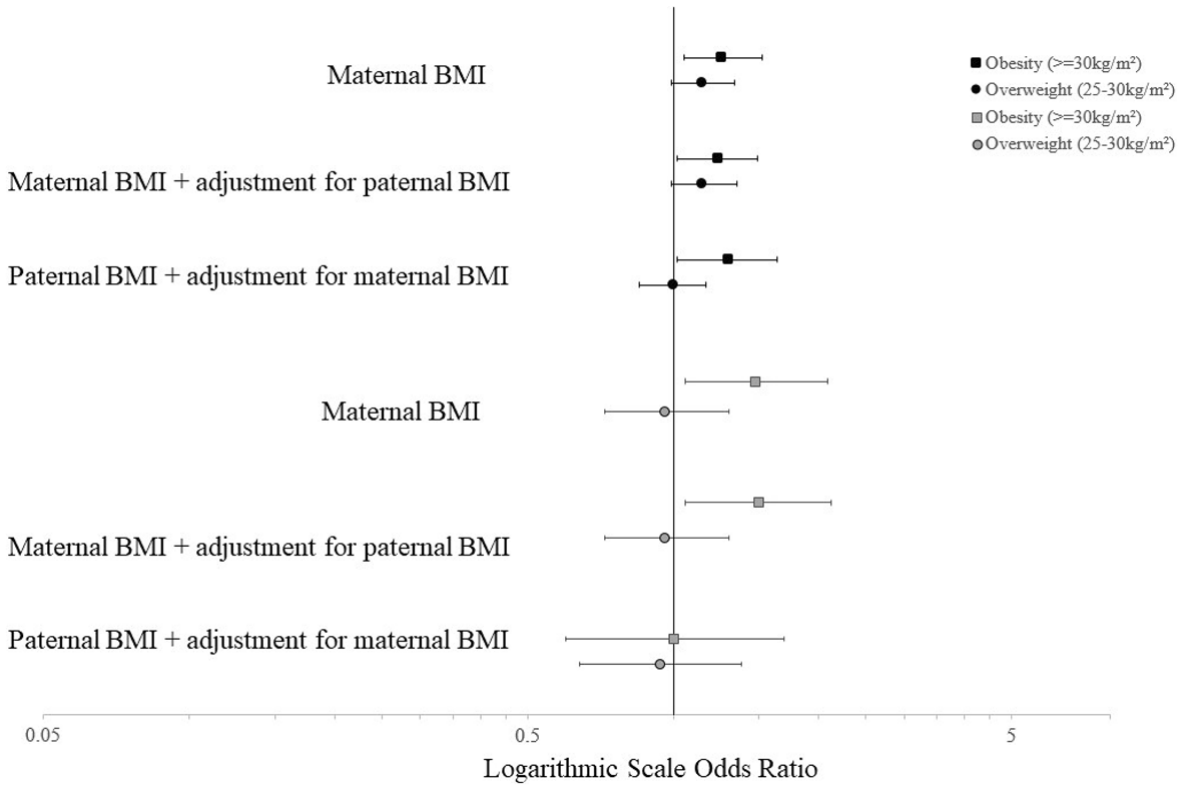
Adjusted odds ratios for a high hyperactivity-inattention symptom score (≥ 7) in 5.5 year old children of the ELFE (n = 10,898; black points) and EPIPAGE 2 cohorts (n = 2646; grey points). ^a^Adjusted for maternal age at birth, maternal education, household income (ELFE), household socioeconomic category (EPIPAGE 2), parity, sex, psychological problems during pregnancy (ELFE), anxiety symptoms (EPIPAGE 2), singleton pregnancy (EPIPAGE 2), maternal physical activity during pregnancy (ELFE), maternal healthy diet score during pregnancy (ELFE), maternal alcohol intake during pregnancy (ELFE), maternal smoking during pregnancy, child age at evaluation, gestational age, breastfeeding, cause of prematurity (EPIPAGE 2) childcare at 2 years, screentime at 2 years (ELFE), HOME score (EPIPAGE 2), difficulties falling asleep at 2 years (ELFE) and frequent night waking at 2 years (EPIPAGE 2). ^b^Missing covariates are imputed and weighted using IPW.

In ELFE, when paternal BMI was added to the model with maternal BMI, though the relationship with maternal obesity with offspring HIS did not change (aOR 1.24 [1.02, 1.49]), paternal obesity was also significantly associated with offspring HIS, with similar magnitude (aOR 1.30 [1.02, 1.64]) (Fig. 2). In EPIPAGE 2, the association between maternal obesity and a high HIS score did not significantly change when the model was adjusted for paternal BMI (aOR 1.53 [1.04, 2.24]) and paternal obesity was not associated with HIS symptoms (aOR 1.08 [0.70, 1.27]).

#### Gestational weight gain adjustment and interactions

**Figure 3:**
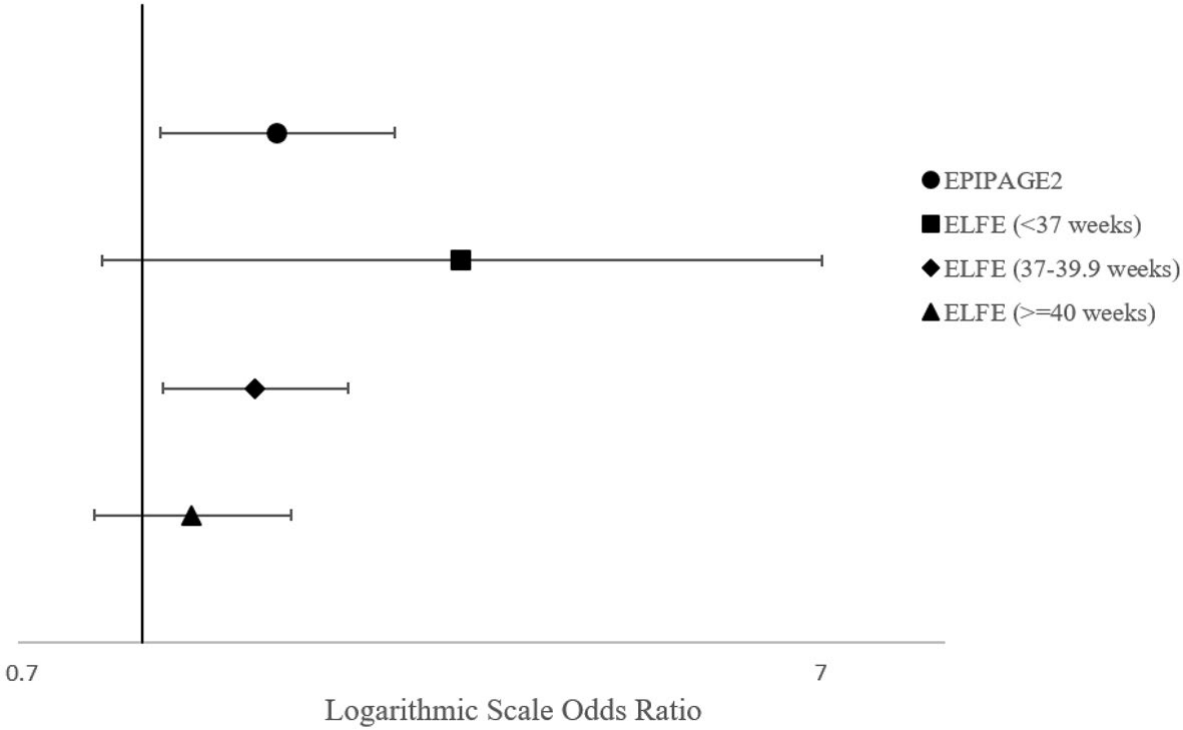
Adjusted Odds ratios for maternal pre-pregnancy obesity and high hyperactivity-inattention symptom scores (≥ 7) by gestational age group in children 5 years old from the ELFE (n = 10,898) and EPIPAGE 2 (n = 2646) cohort studies .^a^Adjusted for maternal age at birth, maternal education, household income (ELFE), household socioeconomic category (EPIPAGE 2), parity, sex, psychological problems during pregnancy (ELFE), anxiety symptoms (EPIPAGE 2), singleton pregnancy (EPIPAGE 2), maternal physical activity during pregnancy (ELFE), maternal healthy diet score during pregnancy (ELFE), maternal alcohol intake during pregnancy (ELFE), maternal smoking during pregnancy, child age at evaluation, gestational age, breastfeeding, cause of prematurity (EPIPAGE 2) childcare at 2 years, screentime at 2 years (ELFE), HOME score (EPIPAGE 2), difficulties falling asleep at 2 years (ELFE) and frequent night waking at 2 years (EPIPAGE 2). ^b^Missing covariates are imputed and weighted using IPW.

Additional adjustment for gestational weight gain did not significantly change the odds ratios between maternal obesity or overweight and a high HIS score in ELFE. A significant interaction between gestational age and maternal BMI was observed in ELFE (*p* = 0.02), but not EPIPAGE 2 (*p* = 0.77). In ELFE, we observed a stronger association between maternal pre-pregnancy obesity and high HIS scores with decreasing gestational age (Fig. 3). Children born preterm (32–37 weeks) had 2.3× increased odds of a high HIS score if born to an obese mother (aOR 2.29 [0.83, 6.33]), while children born between 37 and 39.9 weeks had 1.4× greater odds of a high HIS score (aOR 1.36 [1.04, 1.77]) and children born at full-term (≥ 40 weeks) did not display a significantly increased odds of a high HIS score if born to an obese mother (aOR 1.18 [0.90, 1.56]). None of the other interactions tested were significant (*p* > 0.20).

#### Similar adjustment factors

Using the same adjustment factors in both ELFE and EPIPAGE 2 did not change our estimates from the fully adjusted models (Supplementary Table S6).

## Discussion

Maternal pre-pregnancy obesity was associated with higher HIS scores in offspring at 5.5 years. The magnitude of the association appears to vary by gestational age, with a more pronounced effect in children born preterm. This is the first study to examine this association in a large cohort of preterm infants and to compare the magnitude of the association among gestational age groups. The trends remained after adjustment for paternal BMI, maternal lifestyle variables and factors characterizing the postnatal environment which most previous studies could not account for.

Our results are in accordance with previous studies, which have reported a relationship between HIS and maternal obesity or both obesity and overweight^9^. We also previously reported a positive association between maternal obesity and child HIS trajectories from 3 to 8 years in the French EDEN cohort study^10^. The magnitude of the association in both ELFE and EPIPAGE 2 is somewhat smaller than that of EDEN, where we observed almost two-fold increased odds of a high HIS score trajectory with maternal obesity. Overall, meta-analyses suggest that high maternal pre-pregnancy BMI is associated with child ADHD/HIS^9^. However, several studies have not observed an association between high maternal BMI and child ADHD or HIS^26–28^, and in others the association was not robust among assessment types or evaluators^11,29,30^. To our knowledge, this is the first study to investigate whether the association between maternal obesity and HIS extends to very, moderate and late preterm infants, and to compare the magnitude across a range of gestational ages, including both infants born at term and preterm. In a study of extremely preterm infants (born < 28 weeks), van der Burg et al. found more than 2 × increased odds of parent-reported, but not teacher-reported, HIS in children at 10 years old born to mothers who were obese before pregnancy compared to mothers with a normal pre-pregnancy BMI^11^. This study was severely limited in the potential confounding factors it took into account and it is likely the small sample size also played a role (n < 200). Unfortunately, we did not have enough infants born before 28 weeks to be able to stratify our analyses and our results cannot be directly compared to this study.

In studies considering siblings born at term, positive associations were observed when considering the study population as a whole, but the relationship was attenuated to null in family-level analyses^31,32^, suggesting unmeasured familial confounding may be responsible for the overall association observed in many other studies. In this case, paternal BMI is a valuable negative control, as an association between paternal BMI and child HIS could suggest bias from familial lifestyle, genetics, or shared exposures rather than a direct intrauterine effect of maternal BMI. Indeed, several social factors have been associated with ADHD in offspring such as financial difficulties, social housing tenure and single parent status, which may act through mediators such as parent involvement and adversity risk^33^. However, in contrary to sibling studies, almost all studies adjusting for paternal BMI did not find any association between offspring HIS and paternal BMI^27,34–36^, including our own results in the EPIPAGE 2 or EDEN cohorts^10^. Only Mikkelson et al. observed an association between paternal obesity and child HIS, and observed the greatest behavioural difficulties in children when both parents were overweight or obese, suggesting some influence of the shared familial environment or genetics on the development of HIS^37^. The fact that we observed an association with paternal BMI in ELFE indicates that we cannot attribute the effects on increased HIS solely to an intrauterine mechanism in this study population. A very recent study conducted a Mendelian randomization analysis to test the hypothesis of a causal association between maternal BMI during pregnancy and offspring HIS^38^. The authors observed that the positive association between maternal BMI and offspring HIS remained, though slightly attenuated, after adjusting for both BMI and ADHD polygenic risk scores. This is consistent with the hypothesis that prenatal exposure to high maternal BMI may affect the offspring’s HIS risk through both shared genetic factors and intrauterine mechanisms.

There are several theories of possible biological mechanisms in the relationship between high maternal pre-pregnancy BMI and offspring hyperactivity. Obesity is a state of chronic inflammation and obese women have higher levels of pro-inflammatory factors compared to women with normal weight^4,39^. Proinflammatory cytokines have been linked to oxidative stress and inflammation in the placenta, altered cytokine expression, and changes in fetal brain gene expression^4^. Some cytokine levels, at birth (cord blood) or cross-sectionally at 5 years old, have also been associated with behavioural problems in children^40–42^. In a cross-sectional study of 5-year-old French children, Barbosa et al. found that low levels of the cytokine C–C motif chemokine ligand (CCL)2, responsible for monocyte recruitment to sites of inflammation^43^, were associated with higher HIS scores^41^. However, cord blood cytokines were not associated with HIS scores in children at 5 years^40^. On the other hand, in a follow-up study of infants born extremely preterm (< 28 weeks) previously mentioned, van der Burg et al. found that offspring of overweight or obese mothers were more likely to have increased concentrations of proinflammatory cytokines shortly after birth, but only among infants delivered for maternal or fetal indications and not among infants spontaneously born preterm^44^. As pregnancy disorders related to early spontaneous births are more likely to have inflammatory features than disorders leading to preterm delivery for maternal or fetal indications, they concluded that the role of maternal obesity may be obscured by the inflammation associated with spontaneous indications.

Behavioural development could also be altered by changes to the fetal steroid or hormonal environment due to increased levels of leptin and leptin resistance in obese women^4^. Higher maternal leptin levels have been correlated to higher leptin levels in the placenta, umbilical cord and fetus^4^. Leptin is believed to have a role in fetal brain development, specifically behavioural regulation^45^. In a Japanese cohort, Minatoya et al. observed a positive relationship between maternal pre-pregnancy obesity and high total behavioural problems^46^. In a later study, they unexpectedly found a significant association between increased cord blood leptin and decreased HIS SDQ scores^47^. Future studies are needed to examine the relationship between leptin levels and hyperactivity, as leptin has been shown to vary greatly by ethnicity and could explain the surprising results observed in this Japanese cohort^48^.

However, the effects of maternal pre-pregnancy obesity may not be linked to single metabolites, but rather the effect on the entire maternal metabolome. Girchenko et al. found 43 metabolites were significantly associated with early pregnancy BMI^49^. This profile was significantly associated with higher hazards of any mental and behavioural disorder in children up to 12 years old, as well as higher risk of increasing comorbid disorders^49^. Mediation analyses suggested that up to 60% of the effects of maternal BMI on offspring mental health could be attributed to the metabolomic profile^49^. Further mediation analyses are needed to confirm this potential indirect pathway.

There are several limitations in our study. As both cohorts are observational, there is a possibility that unmeasured confounding remains. We were also not able to adjust for precisely the same factors in both cohorts, which may account for some of the differences in the magnitudes of our estimates. In ELFE, pre-pregnancy weight relied on maternal self-report at delivery and could have caused some measurement error. However, maternal self-reported weight has been found to correlate highly with clinically assessed pre-pregnancy weight^50^. In addition, pre-pregnancy weight is more likely to be under-reported and thus would have attenuated our estimates rather than inflate them^51^. Symptoms of hyperactivity-inattention relied on parental report rather than clinical diagnoses. These scores may reflect biases that vary by evaluator, ethnicity, gender, or socioeconomic factors, but adjustment for maternal country of birth yielded similar results and the interaction between country of birth and SDQ scores was not significant. The SDQ has also been found to be a valid and reliable tool and we used a French-specific threshold for a probable clinical diagnosis score^20,21^. Finally, it’s possible that we observed a smaller risk of HIS symptoms in preterm infants than is true due to the competing risk of neonatal death. High pre-pregnancy BMI is associated with greater risk of perinatal death^25^ and descriptive analyses support this phenomenon in EPIPAGE 2. By conditioning on both live birth and survival to 5 years, the association between maternal obesity and offspring HIS may be distorted and diminished in preterm infants^52^.

This is the first large study of the effect of maternal pre-pregnancy BMI on the development of HIS symptoms in preterm and term infants. Other study strengths include the longitudinal follow-up of two large, nationwide cohort studies. We were able to improve efficiency and reduce bias due to missing data and loss to follow-up using multiple imputation and inverse probability weighting. This is also one of the few studies with available data on important confounders such as maternal diet, physical activity, alcohol consumption, child sleeping patterns and home stimulation. We also had data on paternal BMI available to allow us to use it as a negative control.

## Conclusion

Maternal pre-pregnancy obesity was associated with increased odds of a high hyperactivity-inattention symptom score in both preterm and term children around 5 years of age. The magnitude of the association appears to increase with decreasing gestational age. Paternal BMI was also associated with child HIS scores in ELFE, suggesting that not all of the observed effect can be attributable solely to intrauterine programming due to maternal obesity. Given the growing proportion of obesity in pregnancy, future studies should focus on elucidating possible biological mechanisms involved, such as inflammation and leptin signaling. Efforts should be strengthened to ensure healthy weights in women of child bearing age, and health-care providers should be made aware of the possible increased risk of hyperactivity in the offspring of obese women in order to provide more timely management of the disorder.

## Supplementary Information

Supplementary Information.

## Data Availability

The datasets generated during and analysed during the current study are not publicly available due to privacy laws set by the *Commission nationale de l’informatique et des libertés* (CNIL). Anonymized data may be made available upon reasonable request to any public or private research team and with permission of the ELFE and EPIPAGE 2 scientific committees. Data requests concerning ELFE can be made through the website: https://pandora-elfe.inserm.fr/public/index.php. Data requests concerning EPIPAGE 2 can be made using through the email: accesdonnees.epipage@inserm.fr by using information provided on the data access website: https://epipage2.inserm.fr/index.php/en/related-research/265-data-access-and-questionnaires.

## References

[1] Chen C, Xu X, Yan Y (2018). Estimated global overweight and obesity burden in pregnant women based on panel data model. PLoS ONE.

[2] Leddy MA, Power ML, Schulkin J (2008). The impact of maternal obesity on maternal and fetal health. Rev. Obstet. Gynecol..

[3] Godfrey KM (2017). Influence of maternal obesity on the long-term health of offspring. Lancet Diabetes Endocrinol..

[4] Edlow AG (2017). Maternal obesity and neurodevelopmental and psychiatric disorders in offspring. Prenat. Diagn..

[5] Sayal K, Prasad V, Daley D, Ford T, Coghill D (2018). ADHD in children and young people: Prevalence, care pathways, and service provision. Lancet Psychiatry.

[6] Harpin V (2005). The effect of ADHD on the life of an individual, their family, and community from preschool to adult life. Arch. Dis. Child.

[7] Harpin V, Mazzone L, Raynaud JP, Kahle J, Hodgkins P (2016). Long-term outcomes of ADHD: A systematic review of self-esteem and social function. J. Atten. Disord..

[8] Shook LL, Kislal S, Edlow AG (2020). Fetal brain and placental programming in maternal obesity: A review of human and animal model studies. Prenat. Diagn..

[9] Jenabi E, Bashirian S, Khazaei S, Basiri Z (2019). The maternal prepregnancy body mass index and the risk of attention deficit hyperactivity disorder among children and adolescents: A systematic review and meta-analysis. Korean J. Pediatr..

[10] Dow C, Galera C, Charles M-A, Heude B (2022). Maternal pre-pregnancy BMI and offspring hyperactivity–inattention trajectories from 3 to 8 years in the EDEN birth cohort study. Eur. Child. Adolesc. Psychiatry.

[11] van der Burg JW (2017). Maternal obesity and attention-related symptoms in the preterm offspring. Early Hum. Dev..

[12] Franz AP (2018). Attention-deficit/hyperactivity disorder and very preterm/very low birth weight: A meta-analysis. Pediatrics.

[13] Pierrat V (2021). Neurodevelopmental outcomes at age 5 among children born preterm: EPIPAGE-2 cohort study. The BMJ.

[14] Johnson S, Marlow N (2017). Early and long-term outcome of infants born extremely preterm. Arch. Dis. Child.

[15] Oldenburg KS, O’Shea TM, Fry RC (2020). Genetic and epigenetic factors and early life inflammation as predictors of neurodevelopmental outcomes. Semin. Fetal. Neonatal Med..

[16] Sharma A, Jen R, Butler A, Lavoie P (2012). The developing human preterm neonatal immune system: A case for more research in this area. Clin. Immunol..

[17] Cnattingius S (2013). Maternal obesity and risk of preterm delivery. JAMA.

[18] Charles MA (2021). Cohort Profile: The French national cohort of children (ELFE): Birth to 5 years. Int. J. Epidemiol..

[19] Lorthe E (2021). Cohort Profile: The Etude Epidémiologique sur les Petits Ages Gestationnels-2 (EPIPAGE-2) preterm birth cohort. Int. J. Epidemiol..

[20] Goodman R (2001). Psychometric properties of the strengths and difficulties questionnaire. J. Am. Acad. Child Adolesc. Psychiatry.

[21] Shojaei T, Wazana A, Pitrou I, Kovess V (2009). The strengths and difficulties questionnaire: Validation study in French school-aged children and cross-cultural comparisons. Soc. Psychiatry Psychiatr. Epidemiol..

[22] Kadawathagedara M (2017). Adéquation des consommations alimentaires des femmes enceintes de l’étude ELFE aux recommandations du Programme national nutrition santé. Cahiers de Nutrition et de Diététique.

[23] Bradley RH, Caldwell BM, Corwyn RF (2003). The Child Care HOME Inventories: Assessing the quality of family child care homes. Early Child Res. Q..

[24] Nguyen CD, Carlin JB, Lee KJ (2017). Model checking in multiple imputation: An overview and case study. Emerg. Themes Epidemiol..

[25] Marchi J, Berg M, Dencker A, Olander EK, Begley C (2015). Risks associated with obesity in pregnancy, for the mother and baby: A systematic review of reviews. Obes. Rev..

[26] Brion M-J (2011). Maternal pre-pregnancy overweight and child cognition and behavior: Exploring intrauterine effects in two pregnancy cohorts. Pediatrics.

[27] Casas M (2017). Maternal pre-pregnancy obesity and neuropsychological development in pre-school children: A prospective cohort study. Pediatr. Res..

[28] Li M (2016). The association of maternal obesity and diabetes with autism and other developmental disabilities. Pediatrics.

[29] Jo H (2015). Maternal prepregnancy body mass index and child psychosocial development at 6 years of age. Pediatrics.

[30] Mina TH (2017). Prenatal exposure to maternal very severe obesity is associated with impaired neurodevelopment and executive functioning in children. Pediatr. Res..

[31] Chen Q (2014). Maternal pre-pregnancy body mass index and offspring attention deficit hyperactivity disorder: A population-based cohort study using a sibling-comparison design. Int. J. Epidemiol..

[32] Musser ED (2017). Maternal prepregnancy body mass index and offspring attention-deficit/hyperactivity disorder: A quasi-experimental sibling-comparison, population-based design. J. Child Psychol. Psychiatry.

[33] Russell A, Ford T, Russell G (2015). Socioeconomic associations with ADHD: Findings from a mediation analysis. PLoS ONE.

[34] Daraki V (2017). Effect of parental obesity and gestational diabetes on child neuropsychological and behavioral development at 4 years of age: The Rhea mother–child cohort, Crete, Greece. Eur. Child Adolesc. Psychiatry.

[35] Brion MJ (2011). Intrauterine effects of maternal prepregnancy overweight on child cognition and behavior in 2 cohorts. Pediatrics.

[36] Robinson SL (2020). Parental weight status and offspring behavioral problems and psychiatric symptoms. J. Pediatr..

[37] Mikkelsen SH (2017). Parental body mass index and behavioral problems in their offspring: A Danish national birth cohort study. Am. J. Epidemiol..

[38] Karhunen V (2021). The link between attention deficit hyperactivity disorder (ADHD) symptoms and obesity-related traits : Genetic and prenatal explanations. Transl. Psychiatry.

[39] Rivera HM, Christiansen KJ, Sullivan EL (2015). The role of maternal obesity in the risk of neuropsychiatric disorders. Front Neurosci..

[40] Barbosa S (2020). Immune activity at birth and later psychopathology in childhood. Brain Behav. Immun. Health.

[41] Barbosa S (2020). Serum cytokines associated with behavior: A cross-sectional study in 5-year-old children. Brain Behav. Immun..

[42] Anand D, Colpo GD, Zeni G, Zeni CP, Teixeira AL (2017). Attention-deficit/hyperactivity disorder and inflammation: What does current knowledge tell us? A Systematic Review. Front Psychiatry.

[43] Bose S, Cho J (2013). Role of chemokine CCL2 and its receptor CCR2 in neurodegenerative diseases. Arch. Pharm. Res..

[44] van der Burg JW (2016). The role of systemic inflammation linking maternal BMI to neurodevelopment in children. Pediatr. Res..

[45] Valleau JC, Sullivan EL (2014). The impact of leptin on perinatal development and psychopathology. J. Chem. Neuroanat..

[46] Minatoya M (2017). Associated factors of behavioural problems in children at preschool age: The Hokkaido study on environment and children’s health. Child Care Health Dev..

[47] Minatoya M (2018). Association between fetal adipokines and child behavioral problems at preschool age: The hokkaido study on environment and children’s health. Int. J. Environ. Res. Public Health.

[48] Mente A (2010). Ethnic variation in adiponectin and leptin levels and their association with adiposity and insulin resistance. Diabetes Care.

[49] Girchenko P (2022). Maternal early-pregnancy body mass index-associated metabolomic component and mental and behavioral disorders in children. Mol. Psychiatry.

[50] Lederman SA, Paxton A (1998). Maternal reporting of prepregnancy weight and birth outcome: Consistency and completeness compared with the clinical record. Matern. Child Health J..

[51] Headen I, Cohen AK, Mujahid M, Abrams B (2017). The accuracy of self-reported pregnancy-related weight: A systematic review. Obes. Rev..

[52] Kramer MS, Zhang X, Platt RW (2014). Analyzing risks of adverse pregnancy outcomes. Am. J. Epidemiol..

