# Supplementary material for: Maternal pre-pregnancy obesity and offspring hyperactivity-inattention symptoms at 5 years in preterm and term children: a multi-cohort analysis
Source: Sci Rep. 2022 Oct 28;12:18190. doi: 10.1038/s41598-022-22750-8 (PMC9616941; doi:10.1038/s41598-022-22750-8)
Supplement: Supplementary file 1 — Supplementary Information. [file 41598_2022_22750_MOESM1_ESM.docx]

**Maternal pre-pregnancy obesity and offspring hyperactivity-inattention symptoms at 5 years in preterm and term children: a multi-cohort analysis**

Courtney Dow*^1^, Elsa Lorthe^1,2^, Laetitia Marchand-Martin^1^, Cédric Galera^3,4,5^, Muriel Tafflet^1^, Pierre-Yves Ancel^1^, Marie-Aline Charles^1^, Barbara Heude^1^

^1^Université Paris Cité, Inserm, INRAE, Centre for Research in Epidemiology and StatisticS (CRESS), F-75004 Paris, France

^2^Unit of Population Epidemiology, Department of Primary Care Medicine, Geneva University Hospitals, 1205, Geneva, Switzerland

^3^Univ. Bordeaux, Inserm, Bordeaux Population Health Center, UMR 1219, F-33000, Bordeaux, France

^4^Centre Hospitalier Perrens, Bordeaux, France

^5^Unit on Children’s Psychosocial Maladjustment, Montreal, QC, Canada

**Corresponding Author**: Courtney DOW

courtney.dow@inserm.fr

**Journal**: Scientific Reports

**Supplementary Table S1a**. Missing data in the ELFE cohort study (n=18 329)

| **Variable** | **N** | **Missing (%)** |
| --- | --- | --- |
| *Sociodemographic* |  |  |
| Maternal age (at birth) | 18133 | 1.1 |
| Paternal age (at birth) | 17342 | 5.4 |
| Maternal Country of Birth | 18189 | 0.8 |
| Paternal Country of Birth | 17475 | 4.7 |
| Region of Habitation (Birth) | 18267 | 0.3 |
| Maternal Education (Birth) | 18218 | 0.6 |
| Paternal Education (Birth) | 13894 | 24.2 |
| Maternal Employment Status (Birth) | 18119 | 1.1 |
| Paternal Employment Status (Birth) | 17787 | 3.0 |
| Monthly Income (Birth) | 15543 | 15.2 |
| Cohabitation with Partner (Birth) | 18045 | 1.5 |
|  |  |  |
| *Parental Characteristics* |  |  |
| Parity | 17957 | 2.0 |
| Maternal BMI Category | 17900 | 2.3 |
| Paternal BMI Category | 12653 | 31.0 |
| Maternal Smoking in Pregnancy | 17998 | 1.8 |
| Maternal Smoking before Pregnancy | 17894 | 2.4 |
| Maternal Alcohol Intake in Pregnancy | 18121 | 1.1 |
| Maternal Pre-pregnancy Alcohol Intake | 18128 | 1.1 |
| Maternal Diet Quality Score in Pregnancy | 15467 | 15.6 |
| Maternal Physical Activity Score in Pregnancy | 15532 | 15.3 |
|  |  |  |
| *Maternal Health-Related Characteristics* |  |  |
| Duration of Any Breastfeeding | 15128 | 17.5 |
| Gestational Weight Gain (kg) | 17773 | 3.0 |
| History of Diabetes | 17697 | 3.4 |
| Gestational Diabetes | 17305 | 5.6 |
| Hypertension during Pregnancy | 17701 | 3.4 |
| Pre-eclampsia | 17701 | 3.4 |
| Any Psychiatric Disorder (before pregnancy) | 16975 | 7.4 |
| Any Psychiatric Disorder (during pregnancy) | 18025 | 1.7 |
| Post-Partum Depression | 15751 | 14.1 |
| Folic Acid Intake >12 Weeks | 16926 | 7.7 |
| Folic Acid 0-12 Weeks | 17100 | 6.7 |
| Folic Acid Before Conception | 16808 | 8.3 |
|  |  |  |
| *Child Characteristics* |  |  |
| Sex of Infant | 17747 | 3.2 |
| Gestational Age (days) | 17651 | 3.7 |
| Birth Weight (g) | 17546 | 4.3 |
| Mode of Delivery | 17735 | 3.2 |
| Type of Birth | 17982 | 1.9 |
| Total Screentime (hours/day) (2Y) | 13357 | 27.1 |
| Childcare at 2Y | 13253 | 27.7 |
| Child's Total Sleep Duration/Day (h) at 2Y | 12763 | 30.4 |
| Difficulties Falling Asleep (2Y) | 12933 | 29.4 |
| Child's Age at 5Y Follow-Up | 11439 | 37.6 |
| SDQ Hyperactivity-Inattention Score | 11247 | 38.6 |

**Supplementary Table S1b**. Missing data in the EPIPAGE 2 cohort study (n=4467)

| **Variable** | **N** | **Missing (%)** |
| --- | --- | --- |
| *Sociodemographic* |  |  |
| Maternal Age (Years) | 4467 | 0 |
| Paternal Age (Years) | 2773 | 37.9 |
| Maternal Country of Birth | 4421 | 1.0 |
| Paternal Country of Birth | 3362 | 24.7 |
| Region of Birth | 4467 | 0 |
| Maternal Education (Birth) | 4127 | 7.6 |
| Paternal Level of Education (1Y) | 3335 | 25.3 |
| Maternal Employment Status (Birth) | 4343 | 2.8 |
| Paternal Employment Status (Birth) | 4146 | 7.2 |
| Household SES (Birth) | 4223 | 5.5 |
| Monthly Household Income (Birth) | 3108 | 30.4 |
| Financial Difficulties for Essentials (Birth) | 3393 | 24.0 |
| Living with Spouse or Partner (Birth) | 3421 | 23.4 |
|  |  |  |
| *Parental Characteristics* |  |  |
| Parity | 4414 | 1.2 |
| Maternal BMI Category | 4116 | 7.9 |
| Paternal BMI Category | 1569 | 64.9 |
| Maternal Average Number of Cigarettes/Day | 4265 | 4.5 |
| Paternal Smoking during Pregnancy | 3367 | 24.6 |
| History of Alcoholism, Drug Use or Cannabis | 4410 | 1.3 |
|  |  |  |
| *Maternal Health-Related Characteristics* |  |  |
| Hypertension | 4167 | 6.7 |
| Pre-Eclampsia or Hypertension during Previous Pregnancy | 2399 | 46.3 |
| Permanent Hypertension | 4409 | 1.3 |
| Pre-Eclampsia | 4347 | 2.7 |
| Any Diabetes during Pregnancy | 4111 | 8.0 |
| Gestational Diabetes | 4138 | 7.4 |
| Immunosuppressive Treatment during Pregnancy | 4346 | 2.7 |
| History of Mental Illness | 4406 | 1.4 |
| Folic Acid Supplements around Conception | 3354 | 24.9 |
| Any Breastfeeding | 3574 | 20.0 |
| Anxiety Score (STAI-T) | 2650 | 40.7 |
|  |  |  |
| *Pregnancy Characteristics* |  |  |
| Cause of Prematurity | 4065 | 9.0 |
| Type of Birth (singleton/twins) | 4467 | 0 |
| Type of Delivery | 4419 | 1.1 |
| Bacterial Infection at/during Hospitalisation | 4336 | 2.9 |
| PPROM (≥12h) | 4405 | 1.4 |
| Premature Membrane Rupture Gestational Age* | 4456 | 0.2 |
| Antenatal Corticotherapy | 4380 | 1.9 |
| Histological Chorioamnionitis | 4373 | 2.1 |
| CRP: Last Results before Delivery (mg/l)* | 2859 | 36.0 |
|  |  |  |
| *Child Characteristics* |  |  |
| Child Sex | 4467 | 0 |
| Gestational Age (Weeks) | 4467 | 0 |
| Birth Weight (g) | 4467 | 0 |
| <10th Percentile According to EPOPE Curves | 4466 | 0.0 |
| Severe Neonatal Morbidity | 4239 | 5.1 |
| Childcare at 2Y | 3610 | 19.2 |
| Difficulty Falling Asleep (2Y) | 3557 | 20.4 |
| Child Wakes Up (2y) | 3541 | 20.7 |
| First Language | 3412 | 23.6 |
| Corrected Age at 5Y Follow-Up (Parent Questionnaire) (Months) | 3018 | 32.4 |
| Difficulty Falling Asleep (5Y) | 2992 | 33.0 |
| Child Wakes Up (5Y) | 2953 | 33.9 |
| SDQ Hyperactivity Score | 2648 | 40.7 |
| HOME Score | 2519 | 43.6 |

^a^Only asked for specific clinical situations such as preterm labour or PPROM

^b^BMI: body mass index; CRP: C-reactive protein; EPOPE: Obstetrical, Perinatal and Pediatric Epidemiology research team at INSERM (French Institute for Health and Medical Research); IUGR: intrauterine growth restriction; HOME: Home Observation Measurement of the Environment-Short Form

**Supplementary Table S2a**. Descriptive characteristics (N [%] or mean [STD]) of the population lost to follow-up at 5.5 years in the ELFE study (n=18 270)

|  | | **Follow-up** | |  |
| --- | --- | --- | --- | --- |
|  | **Yes** | **No** | **P-value^1^** |  |
| **Variable** |  | **n=11 184 (%)** | **n=7086 (%)** |  |
| *Sociodemographics* |  |  |  |  |
| Maternal Age at Child's Birth |  | 30.9 (4.7) | 29.1 (5.5) | <0.001 |
| Maternal Country of Birth | France | 10173 (90.5) | 5694 (81.1) | <0.001 |
|  | EU | 240 (2.1) | 167 (2.4) |  |
|  | Other | 834 (7.4) | 1162 (16.5) |  |
| Maternal Education (Birth) | High | 7628 (67.8) | 2720 (38.7) | <0.001 |
|  | Medium | 3119 (27.7) | 3243 (46.2) |  |
|  | Low | 500 (4.4) | 1060 (15.1) |  |
| Paternal Education (Birth) | High | 6238 (55.5) | 2566 (36.5) | <0.001 |
|  | Medium | 4236 (37.7) | 3612 (51.4) |  |
|  | Low | 773 (6.9) | 845 (12.0) |  |
| Maternal Employment | Employed/Self-Employed | 9705 (86.3) | 4630 (65.9) | <0.001 |
|  | Unemployed/Student | 750 (6.7) | 866 (12.3) |  |
|  | Domestic tasks/inactive/other | 792 (7.0) | 1527 (21.7) |  |
| Paternal Employment | Employed/Self-Employed | 10417 (92.6) | 5880 (83.7) | <0.001 |
|  | Unemployed/Student | 753 (6.7) | 842 (12.0) |  |
|  | Domestic tasks/inactive/other | 77 (0.7) | 301 (4.3) |  |
| Monthly Revenue | 1st Quartile | 2828 (25.1) | 3451 (49.1) | <0.001 |
|  | 2nd Quartile | 3873 (34.4) | 2073 (29.5) |  |
|  | 3rd Quartile | 2705 (24.1) | 922 (13.1) |  |
|  | 4th Quartile (Highest) | 1841 (16.4) | 577 (8.2) |  |
| Cohabitation with Partner (Birth) | Yes | 10925 (97.1) | 6339 (90.3) | <0.001 |
|  | No | 322 (2.9) | 684 (9.7) |  |
| Parity | 0 | 5129 (46.2) | 3120 (45.5) | <0.001 |
|  | 1 | 4036 (36.4) | 2211 (32.2) |  |
|  | 2 | 1450 (13.1) | 936 (13.7) |  |
|  | ≥3 | 485 (4.4) | 590 (8.6) |  |
|  |  |  |  |  |
| *Pregnancy/Delivery* |  |  |  |  |
| Type of Birth | Singleton | 10898 (96.9) | 6798 (96.8) | 0.7 |
|  | Twins | 349 (3.1) | 225 (3.2) |  |
| Mode of Delivery | Vaginal (Normal) | 7598 (69.3) | 4574 (67.5) | <0.001 |
|  | Forceps, vacuum | 1406 (12.8) | 830 (12.3) |  |
|  | Cesarean | 1956 (17.8) | 1371 (20.2) |  |
| Sex of Infant | Male | 5827 (51.8) | 3555 (50.6) | 0.12 |
|  | Female | 5420 (48.2) | 3468 (49.4) |  |
| Gestational Age (days) |  | 276.8 (10.5) | 276.7 (10.4) | 0.43 |
| Birth Weight (g) |  | 3312.2 (499.4) | 3301.6 (494.9) | 0.16 |
|  |  |  |  |  |
| *Parental Health Characteristics* |  |  |  |  |
| Maternal BMI Category | Underweight (<18.5kg/m²) | 784 (7.1) | 622 (9.1) | <0.001 |
|  | Normal (18.5-25kg/m²) | 7489 (67.5) | 4126 (60.6) |  |
|  | Overweight (25-30kg/m²) | 1828 (16.5) | 1255 (18.4) |  |
|  | Obese (≥30kg/m²) | 994 (9.0) | 802 (11.8) |  |
| Paternal BMI Category | Underweight (<18.5kg/m²) | 75 (0.8) | 20 (0.7) | <0.01 |
|  | Normal (18.5-25kg/m²) | 5374 (55.5) | 1587 (53.5) |  |
|  | Overweight (25-30kg/m²) | 3500 (36.1) | 1075 (36.2) |  |
|  | Obese (≥30kg/m²) | 737 (7.6) | 285 (9.6) |  |
| Folic Acid Before Conception | No | 8622 (76.7) | 6073 (86.5) | <0.001 |
|  | Yes | 2625 (23.3) | 950 (13.5) |  |
| Folic Acid 0-12 Weeks | No | 7888 (70.1) | 5674 (80.8) | <0.001 |
|  | Yes | 3359 (29.9) | 1349 (19.2) |  |
| Folic Acid Intake >12 Weeks | No | 8642 (76.8) | 5637 (80.3) | <0.001 |
|  | Yes | 2605 (23.2) | 1386 (19.7) |  |
| Gestational Weight Gain (kg) |  | 13.2 (5.2) | 13.2 (6.1) | 0.63 |
| Duration of Any Breastfeeding | None | 3636 (32.3) | 2363 (33.6) | 0.18 |
|  | <6 months | 5348 (47.6) | 3281 (46.7) |  |
|  | >=6 months | 2263 (20.1) | 1379 (19.6) |  |
| History of Diabetes | No | 10821 (96.2) | 6707 (95.5) | 0.04 |
|  | Type I/Type II | 107 (1.0) | 70 (1.0) |  |
|  | Previous Gestational Diabetes | 319 (2.8) | 246 (3.5) |  |
| Gestational Diabetes | No | 10487 (93.2) | 6491 (92.4) | 0.04 |
|  | Yes | 760 (6.8) | 532 (7.6) |  |
| Hypertension during Pregnancy | No | 11021 (98.0) | 6875 (97.9) | 0.65 |
|  | Yes | 226 (2.0) | 148 (2.1) |  |
| Pre-eclampsia | No | 11095 (98.6) | 6908 (98.4) | 0.12 |
|  | Yes | 152 (1.4) | 115 (1.6) |  |
| Any Psychiatric Disorder (before pregnancy) | No | 11204 (99.6) | 6976 (99.3) | <0.01 |
|  | Yes | 43 (0.4) | 47 (0.7) |  |
| Any Psychiatric Disorder (during pregnancy) | No | 9875 (87.8) | 6099 (86.8) | 0.06 |
|  | Yes | 1372 (12.2) | 924 (13.2) |  |
| Post-Partum Depression | No | 10261 (91.2) | 6233 (88.8) | <0.001 |
|  | Yes | 986 (8.8) | 790 (11.2) |  |
|  |  |  |  |  |
| *Maternal Lifestyle* |  |  |  |  |
| Maternal Smoking before Pregnancy | None | 6644 (59.1) | 3965 (56.5) | <0.001 |
|  | <10/day | 1834 (16.3) | 973 (13.9) |  |
|  | >=10/day | 2769 (24.6) | 2085 (29.7) |  |
| Maternal Smoking in Pregnancy | No | 9385 (83.4) | 5214 (74.2) | <0.001 |
|  | Yes | 1862 (16.6) | 1809 (25.8) |  |
| Maternal Alcohol Intake in Pregnancy | Light (<3 units) | 6768 (60.2) | 5562 (79.2) | <0.001 |
|  | Moderate (3-7 units) | 4384 (39.0) | 1401 (19.9) |  |
|  | Heavy (≥7 units) | 95 (0.8) | 60 (0.9) |  |
| Diet Quality Score in Pregnancy |  | 7.7 (0.8) | 7.4 (0.8) | <0.001 |
| Physical Activity Score |  | 174.8 (85.2) | 186.8 (102.3) | <0.001 |

^1^Difference by attrition, by ANOVA or by chi² test

**Supplementary Table S2b**. Descriptive characteristics of the population (N [%] or mean [STD]) lost to follow-up at 5.5 years in the EPIPAGE 2 study (n=4467)

|  | | | | | |
| --- | --- | --- | --- | --- | --- |
|  | | **Follow-Up** | |  |  |
| **Variable** |  | **Yes (n=2648)** | **No (n=1819)** | **P-value^1^** |  |
| *Sociodemographics* |  |  |  |  |  |
| Maternal Age (Years) |  | 30.6 [0.14] | 29.3 [0.18] | <0.001 |  |
| Maternal Country of Birth | France | 2169 (84.7) | 1263 (73.0) | <0.001 |  |
|  | Africa | 314 (9.9) | 327 (15.6) |  |  |
|  | Other | 157 (5.3) | 191 (11.4) |  |  |
| Maternal Education (Birth) | High | 1351 (54.0) | 530 (35.9) | <0.001 |  |
|  | Medium | 528 (20.4) | 363 (23.5) |  |  |
|  | Low | 690 (25.6) | 665 (40.6) |  |  |
| Husband/Partner Level of Education (1Y) | High | 1001 (44.6) | 325 (34.0) | <0.001 |  |
|  | Medium | 522 (21.2) | 247 (22.7) |  |  |
|  | Low | 768 (34.2) | 472 (43.3) |  |  |
| Maternal Employment Status (Birth) | Employed | 1816 (71.8) | 958 (58.1) | <0.001 |  |
|  | Unemployed | 245 (9.2) | 212 (10.6) |  |  |
|  | Student/Apprentice | 61 (2.2) | 54 (2.7) |  |  |
|  | Domestic Tasks | 382 (13.9) | 433 (23.0) |  |  |
|  | Other | 75 (2.9) | 107 (5.5) |  |  |
| Paternal Employment Status (Birth) | Employed | 2250 (90.0) | 1330 (82.9) | <0.001 |  |
|  | Unemployed | 165 (7.1) | 187 (9.8) |  |  |
|  | Student/Apprentice | 21 (0.6) | 30 (1.9) |  |  |
|  | Domestic Tasks | 10 (0.5) | 18 (0.7) |  |  |
|  | Other | 52 (1.9) | 83 (4.6) |  |  |
| Living with Spouse or Partner | No | 157 (6.9) | 199 (15.8) | <0.001 |  |
|  | Yes | 1990 (93.1) | 1075 (84.2) |  |  |
| Household SES | Managerial | 661 (27.3) | 262 (16.5) | <0.001 |  |
|  | Intermediate | 632 (25.8) | 252 (16.1) |  |  |
|  | Administrator, Company Director, Civil Servant, Student | 647 (25.2) | 506 (30.5) |  |  |
|  | Domestic or Sales Employee | 309 (11.3) | 298 (17.6) |  |  |
|  | Labourer | 249 (8.7) | 265 (14.4) |  |  |
|  | Without Profession | 43 (1.7) | 99 (4.9) |  |  |
| Monthly Household Income | <1000 | 144 (6.9) | 176 (14.2) | <0.001 |  |
|  | 1000-2000 | 430 (19.7) | 334 (28.3) |  |  |
|  | 2000-3000 | 609 (31.5) | 299 (24.7) |  |  |
|  | 3000-4000 | 457 (23.3) | 193 (20.3) |  |  |
|  | ≥4000 | 335 (18.6) | 131 (12.5) |  |  |
| Parity | Primiparous | 1502 (56.8) | 890 (51.6) | <0.001 |  |
|  | 1 | 632 (25.4) | 414 (21.2) |  |  |
|  | 2+ | 488 (17.8) | 488 (27.2) |  |  |
| *Pregnancy/Delivery* |  |  |  |  |  |
| Birth Weight (g) |  | 1712.5 [12.46] | 1751.8 [14.52] | 0.01 |  |
| Gestational Age | ≥32 weeks | 658 (64.2) | 535 (69.4) | <0.001 |  |
|  | 27-31 weeks | 1660 (31.2) | 1063 (26.5) |  |  |
|  | <27 weeks | 330 (4.6) | 221 (4.1) |  |  |
| Child Sex | Male | 1421 (55.5) | 934 (50.4) | 0.01 |  |
|  | Female | 1227 (44.5) | 885 (49.6) |  |  |
| Cause of Prematurity | Preterm labour | 629 (41.8) | 495 (45.4) | 0.63 |  |
|  | PPROM | 385 (24.1) | 287 (23.6) |  |  |
|  | Vascular pathology without IUGR | 225 (13.0) | 156 (13.2) |  |  |
|  | Vascular pathology with IUGR | 233 (11.6) | 127 (9.4) |  |  |
|  | Isolated placental abruption | 48 (3.2) | 32 (2.8) |  |  |
|  | Isolated IUGR | 104 (6.4) | 66 (5.7) |  |  |
| Type of Delivery | Vaginal (Normal) | 826 (33.8) | 640 (40.0) | <0.01 |  |
|  | Instrumental | 99 (5.4) | 69 (4.4) |  |  |
|  | Cesarean | 1696 (60.9) | 1089 (55.6) |  |  |
| Preterm Premature Rupture of Membrane (>12h) | No | 1825 (69.5) | 1203 (66.1) | 0.08 |  |
|  | Yes | 791 (30.5) | 586 (33.9) |  |  |
| Gestational Age at Preterm Premature Rupture of Membrane |  | 31.3 [0.06] | 31.7 [0.06] | <0.001 |  |
| Severe Neonatal Morbidity | No | 2208 (93.0) | 1489 (93.9) | 0.23 |  |
|  | Yes | 325 (7.0) | 217 (6.1) |  |  |
| *Parental Health/Lifestyle* |  |  |  |  |  |
| Maternal BMI Category | Underweight (<18.5kg/m²) | 180 (6.7) | 165 (11.0) | <0.01 |  |
|  | Normal (18.5-25kg/m²) | 1430 (58.8) | 955 (59.2) |  |  |
|  | Overweight (25-30kg/m²) | 488 (20.1) | 290 (17.1) |  |  |
|  | Obese (≥30kg/m²) | 367 (14.3) | 241 (12.7) |  |  |
| Paternal BMI Category | Underweight (<18.5kg/m²) | 5 (0.4) | 8 (1.6) | N/A* |  |
|  | Normal (18.5-25kg/m²) | 545 (56.7) | 320 (53.8) |  |  |
|  | Overweight (25-30kg/m²) | 323 (30.7) | 206 (35.1) |  |  |
|  | Obese (≥30kg/m²) | 102 (12.1) | 60 (9.5) |  |  |
| Folic Acid Supplements around Conception | No | 1324 (64.6) | 884 (70.1) | 0.01 |  |
|  | Yes | 780 (35.4) | 366 (29.9) |  |  |
| Average Number of Cigarettes/Day | Non-Smoker | 2080 (83.7) | 1299 (75.6) | <0.001 |  |
|  | Light Smoker (1-5) | 139 (4.7) | 129 (7.1) |  |  |
|  | Moderate Smoker (6-10) | 227 (8.3) | 208 (11.8) |  |  |
|  | Heavy Smoker (≥11) | 89 (3.3) | 94 (5.5) |  |  |
| Any Diabetes during Pregnancy | No | 2201 (88.2) | 1482 (87.9) | 0.84 |  |
|  | Yes | 251 (11.8) | 177 (12.1) |  |  |
| Hypertension during Pregnancy | No | 2079 (85.3) | 1409 (84.1) | 0.42 |  |
|  | Yes | 412 (14.7) | 267 (15.9) |  |  |
| Anxiety Score (STAI-T) | Weak | 1409 (81.7) | 716 (83.4) | 0.54 |  |
|  | Moderate | 222 (11.0) | 108 (10.8) |  |  |
|  | High | 150 (7.3) | 45 (5.8) |  |  |
| ^a^Difference among BMI categories, by ANOVA or by chi² test | | | | |  |

^b^BMI: body mass index; CRP: C-reactive protein; EPOPE: Obstetrical, Perinatal and Pediatric Epidemiology research team at INSERM (French Institute for Health and Medical Research); IUGR: intrauterine growth restriction; HOME: Home Observation Measurement of the Environment-Short Form

*N/A: Categories too small to perform chi² test

**Supplementary Table S3.** Population characteristics by vital status in the full EPIPAGE 2 cohort study (n=7804)

|  | | **Vital Status** | | | | |  |
| --- | --- | --- | --- | --- | --- | --- | --- |
| **Variable** |  | **Termination of Pregnancy** | **Stillbirth** | **Death in Delivery Room** | **Death in Neonatology** | **Alive at Discharge from Neonatology** | **P-value^1^** |
|  |  | **n=1328** | **n=1306** | **n=290** | **n=413** | **n=4467** |  |
| Maternal Body Mass Index (kg/m²) | | 23.8 [0.2] | 24.8 [0.2] | 24.4 [0.4] | 24.6 [0.4] | 24.2 [0.1] | 0.011 |
| Maternal BMI Category | Underweight (<18.5kg/m²) | 100 (8.2) | 86 (7.4) | 10 (3.8) | 38 (10.9) | 345 (8.5) | 0.015 |
|  | Normal (18.5-25kg/m²) | 716 (61.8) | 575 (55.0) | 148 (63.6) | 189 (51.3) | 2385 (59.0) |  |
|  | Overweight (25-30kg/m²) | 218 (18.3) | 238 (21.6) | 44 (17.0) | 74 (20.2) | 778 (18.8) |  |
|  | Obese (≥30kg/m²) | 129 (11.7) | 174 (16.0) | 41 (15.6) | 56 (17.7) | 608 (13.7) |  |
|  | | | | | | | |

^a^Difference among BMI categories, by ANOVA or by chi² test

**Supplementary Table S4a**. Logistic regression complete case analysis for hyperactivity-inattention symptom scores at 5.5 years in the ELFE cohort study

|  | **Univariate** | **Model 2** | **Model 3** | **Model 4** |
| --- | --- | --- | --- | --- |
|  | **n=10 640** | **n=10 015** | **n=8959** | **n=7185** |
| **Variable** | **Unadjusted OR [95%CI]** | **Adjusted OR^a^ [95%CI]** | **Adjusted OR^b^ [95%CI]** | **Adjusted OR^c^ [95%CI]** |
| Obese (≥30kg/m²) | 1.61 [1.33, 1.94] | 1.43 [1.17, 1.74] | 1.44 [1.17, 1.77] | 1.40 [1.10, 1.78] |
| Overweight (25-30kg/m²) | 1.36 [1.16, 1.60] | 1.22 [1.04, 1.44] | 1.27 [1.07, 1.51] | 1.29 [1.06, 1.57] |
| Normal (18.5-25kg/m²) | REF | REF | REF | REF |
| Underweight (<18.5kg/m²) | 1.11 [0.88, 1.41] | 1.02 [0.79, 1.31] | 1.01 [0.77, 1.32] | 1.06 [0.78, 1.44] |

^a^Adjusted for maternal education, household income, parity, sex, psychological problems during pregnancy, maternal age at birth

^b^Additionally adjusted for maternal physical activity during pregnancy, maternal healthy diet score during pregnancy, maternal alcohol intake during pregnancy, maternal smoking during pregnancy

^c^Additionally adjusted for child age at evaluation, gestational age, breastfeeding, childcare at 2 years, screentime at 2 years, frequent night waking at 2 years

**Supplementary Table S4b.** Complete case analysis using generalized estimating equations for hyperactivity-inattention symptom scores at 5.5 years in the EPIPAGE 2 cohort study

|  | **Univariate** | **Model 2** | **Model 3** | **Model 4** | **Model 5** |
| --- | --- | --- | --- | --- | --- |
|  | **N=2465** | **N=2304** | **N=1598** | **N=1469** | **N=1232** |
| **Variable** | **Unadjusted OR [95%CI]** | **Adjusted OR^a^ [95%CI]** | **Adjusted OR^b^ [95%CI]** | **Adjusted OR^c^ [95%CI]** | **Adjusted OR^d^ [95%CI]** |
| Obese (≥30kg/m²) | 1.50 [1.16, 1.93] | 1.54 [1.17, 2.03] | 1.50 [1.07, 2.11] | 1.38 [0.94, 2.01] | 1.40 [0.93, 2.12] |
| Overweight (25-30kg/m²) | 1.06 [0.83, 1.36] | 1.11 [0.85, 1.45] | 1.17 [0.84, 1.63] | 0.98 [0.68, 1.40] | 0.96 [0.64, 1.46] |
| Normal (18.5-25kg/m²) | REF | REF | REF | REF | REF |
| Underweight (<18.5kg/m²) | 1.10 [0.78, 1.56] | 1.18 [0.83, 1.69] | 1.37 [0.91, 2.06] | 1.51 [0.96, 2.35] | 1.84 [1.10, 3.09] |

^a^Adjusted for maternal education, maternal age at birth, household socioeconomic category, parity, sex, and singleton pregnancy

^b^Additionally adjusted for maternal smoking in pregnancy and symptoms of anxiety

^c^Additionally adjusted for child age at evaluation, gestational age, and cause of prematurity

^d^Additionally adjusted for breastfeeding, HOME score, childcare at 2 years and frequent night waking at 2 years

**Supplementary Table S5**. Linear regression for hyperactivity-inattention symptom scores in the ELFE (n=10 898) and EPIPAGE 2 (n=2646) cohort studies at 5.5 years

| **Variable** | **Unadjusted β [95%CI]** | **Adjusted β^1^ [95%CI]** |
| --- | --- | --- |
| *ELFE* |  |  |
| Obese (≥30kg/m²) | 0.44 [0.28, 0.59] | 0.27 [0.12, 0.43] |
| Overweight (25-30kg/m²) | 0.38 [0.25, 0.51] | 0.25 [0.12, 0.37] |
| Normal (18.5-25kg/m²) | REF | REF |
| Underweight (<18.5kg/m²) | 0.20 [0.02, 0.38] | 0.07 [-0.10, 0.25] |
|  |  |  |
| *EPIPAGE 2* |  |  |
| Obese (≥30kg/m²) | 0.68 [0.32, 1.04] | 0.67 [0.30, 1.04] |
| Overweight (25-30kg/m²) | 0.15 [-0.16, 0.45] | 0.19 [-0.10, 0.48] |
| Normal (18.5-25kg/m²) | REF | REF |
| Underweight (<18.5kg/m²) | 0.38 [-0.14, 0.91] | 0.10 [-0.39, 0.59] |

^a^Adjusted for maternal age at birth, maternal education, household income (ELFE), household socioeconomic category (EPIPAGE 2), parity, sex, psychological problems during pregnancy (ELFE), anxiety symptoms (EPIPAGE 2), singleton pregnancy (EPIPAGE 2), maternal physical activity during pregnancy (ELFE), maternal healthy diet score during pregnancy (ELFE), maternal alcohol intake during pregnancy (ELFE), maternal smoking during pregnancy, child age at evaluation, gestational age, breastfeeding, childcare at 2 years, screentime at 2 years (ELFE), HOME score (EPIPAGE 2), difficulties falling asleep at 2 years (ELFE) and frequent night waking at 2 years (EPIPAGE 2)

**Supplementary Table S6.** Logistic regression for high hyperactivity-inattention scores at 5.5 years in the ELFE (n=10 898) and EPIPAGE 2 (n=2646) cohort studies adjusted for similar covariates

|  | **ELFE** | **EPIPAGE 2** |
| --- | --- | --- |
| **Variable** | **Adjusted OR^a^ [95%CI]** | **Adjusted OR^a^ [95%CI]** |
| Obese (≥30kg/m²) | 1.29 [1.08,1.55] | 1.49 [1.02, 2.16] |
| Overweight (25-30kg/m²) | 1.18 [1.01,1.38] | 0.98 [0.69, 1.40] |
| Normal (18.5-25kg/m²) | REF | REF |
| Underweight (<18.5kg/m²) | 1.13 [0.92,1.41] | 0.88 [0.52, 1.48] |

^a^Adjusted for maternal age at birth, maternal education, household income (ELFE) or household socioeconomic category (EPIPAGE 2), parity, sex, psychological problems during pregnancy (ELFE) or anxiety symptoms (EPIPAGE 2), maternal smoking during pregnancy, child age at evaluation, and gestational age
